# Nanotechnology-Based Weapons to Combat Human Papillomavirus Infection Associated Diseases

**DOI:** 10.3389/fchem.2021.798727

**Published:** 2021-11-17

**Authors:** Luyao Pan, Bingxin Li, Jiahua Chen, Haofeng Zhang, Xi Wang, Jiahui Shou, Dejun Yang, Xiaojian Yan

**Affiliations:** ^1^ Department of Gynecology, the First Affiliated Hospital of Wenzhou Medical University, Wenzhou, China; ^2^ School of Biomedical Engineering, School of Ophthalmology and Optometry and Eye Hospital, Wenzhou Medical University, Wenzhou Institute, University of Chinese Academy of Sciences, Wenzhou, China

**Keywords:** nanotechnology, HPV, detection, treatment, nanomaterials

## Abstract

Persistent human papillomavirus (HPV) infection will eventually lead to clinical problems, varying from verrucous lesions to malignancies like cervical cancer, oral cancer, anus cancer, and so on. To address the aforementioned problems, nanotechnology-based strategies have been applied to detect the virus, prevent the interaction between virus and mammalian cells, and treat the virus-infected cells, due mainly to the unique physicochemical properties of nanoparticles. In this regard, many nanotechnology-based chemotherapies, gene therapy, vaccination, or combination therapy have been developed. In this Minireview, we outline the pathogenesis of HPV infection and the recent advances in nanotechnology-based weapons that can be applied in combating HPV-associated diseases.

## Introduction

Human papillomavirus (HPV) is an envelope-free double-stranded circular DNA virus composed of DNA core and protein capsid. HPV parasitizes on the surface of the genitals and leads to the proliferation of the squamous epithelial cells on human skin and mucous membrane (Franco et al., 1999; Lefkowitz et al., 2018). Most invaded HPV will be cleared by our immune system without any clinical symptoms. However, a persistent and chronic HPV infection may cause diseases (Franco et al., 1999). The clinical manifestations of the HPV persistent infection vary from verrucous lesions to malignancies, which depend on the subtype of the infected virus, as well as the duration of infection. For example, HPV 6, 11, and 32 can lead to the emergence of verruca vulgaris and verruca plana, while HPV 16, 18, 31, 33, and 39 cause cancers of cervix, oral cavity, rectum, vulva, tonsil, etc. (Smith et al., 2007; Howell-Jones et al., 2010). Therefore, to control HPV-associated diseases, techniques that can be used to diagnose, prevent, or treat HPV-induced infections are highly desired.

At present, there are two ways to detect HPV infection, namely serological test and molecular test. The former method needs to be performed in the existence of a viral antigen or antibody, and the reaction between the virus and its antigen or antibody can only be tested in the initial period of virus infection. Moreover, the serological test always leads to a high false-positive rate because of the cross-reactivity of the used antibody. Thus, this method of virus detection has fallen into obsolescence now (Draz and Shafiee, 2018). At the same time, molecular technology represented by polymerase chain reaction (PCR) plays an important role in virus detection, which is performed depending on nucleic acid amplification and is superior in detection speed and sensitivity compared to the serological test. However, this method can only be performed on certain pieces of specialized laboratory equipment, and still has a deficiency in repeatability, accuracy, and specificity due to the high genetic variability of some viruses (Ratcliff et al., 2007; Draz et al., 2014). Regarding the treatment of HPV infections, there are mainly two ways, drug-based or nondrug-based therapies. The commonly used therapeutics include cytotoxic agents (5-fluorouracil, bleomycin, etc), antiviral agents (acyclovir), and immune modulators (interferon-α), and they are promising in treating some mild diseases like verrucous. Nondrug-based therapies include cryotherapy, CO_2_ laser therapy, photodynamic therapy, and surgery. They are used for both mild diseases and precancerous lesions. Although these strategies have been widely used in treating HPV infections, they often suffer from high recurrence rates and have a result in scar hyperplasia or increased infection rate (Soheili et al., 2021). Therefore, novel strategies that can be used to prevent the invasion of HPV infection or to treat HPV infection-associated diseases are of great importance.

**FIGURE 1:**
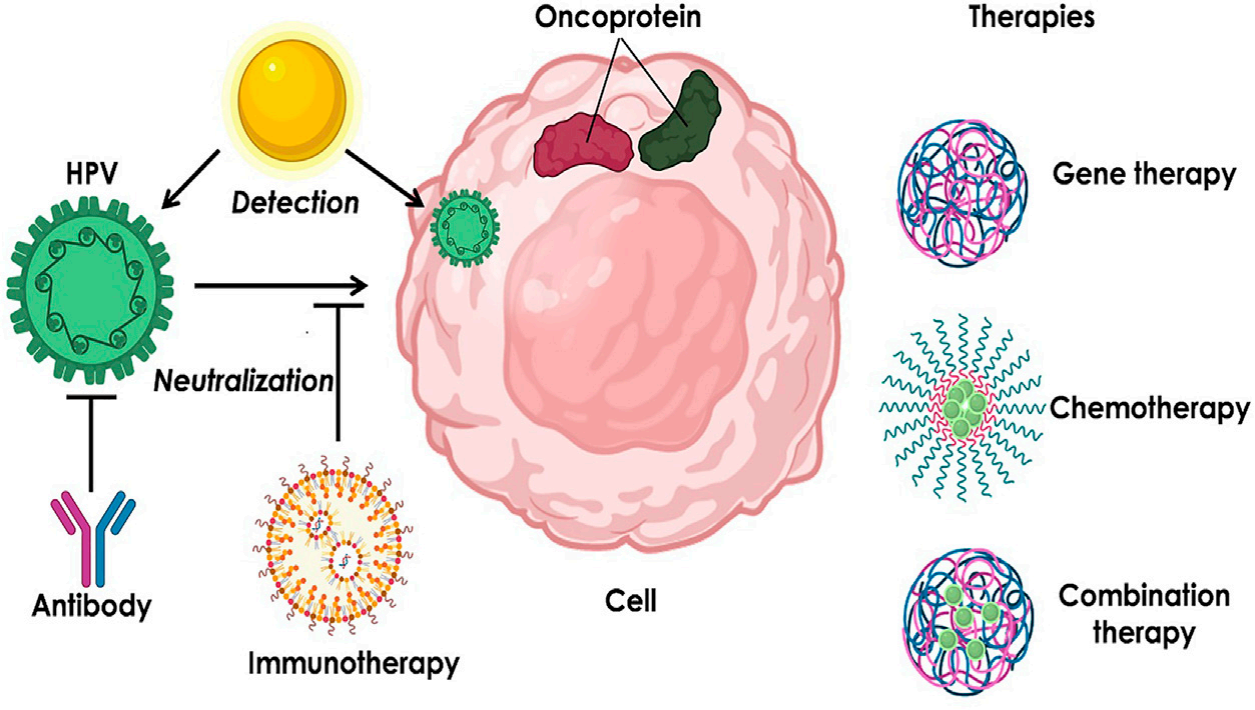
Summary of the nanotechnology-based strategies for HPV infection-induced diseases.

Nanomaterials (including inorganic, organic, polymeric, and hybrid nanoparticles) have been widely developed and applied in biomedicine and other fields since the late 1960s (Wang et al., 2009a; Draz and Shafiee, 2018). Nanomaterials have many unique properties, such as optical, electronic, magnetic, stimuli-responsive properties. Therefore, nanotechnology-based strategies are promising in detecting HPV infections, neutralizing HPV, and treating the diseases caused by HPV infections (Figure 1). In this minireview, we focus on the recent development of technologies that have been used to control HPV-induced diseases. Also, we highlight the remaining issues to be addressed in future work to translate the nanomedicine from bench to bedside.

## The Pathogenesis of HPV Infection

**FIGURE 2:**
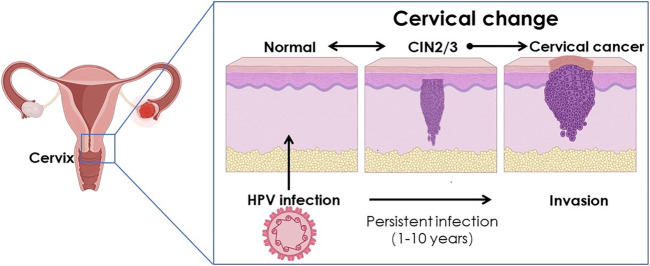
Schematic illustration of the cervical change caused by HPV infection.

Several studies have elaborated on the role of inflammation in the initiation and development of cancers, as illustrated in Figure 2. Many crucial factors have been identified, including key inflammatory factors, tumor-infiltrating leukocytes, the association of cancer-related inflammation with adaptive immunity or sex hormones, etc. (Mantovani et al., 2008). However, the role of inflammation in HPV infection and HPV-associated cervical cancer remains largely undiscovered. It was not until recently that Javid Sadri Nahand *et al.* described the pathogenic role of exosomes and micro ribonucleic acids (miRNAs) in HPV-mediated inflammation.

Exosomes are a group of nanovesicles with sizes ranging from 30 to 150 nm (Alenquer and Amorim, 2015; Helwa et al., 2017). Exosomes play an important role in intercellular communication by transferring nucleic acids, proteins, and lipids from donor cells to recipient cells (Li et al., 2013; Mirzaei et al., 2017). The functions of exosomes mentioned above also exist in HPV infected cells, one of the executors of which is a substance called extracellular survivin. Early in 2009, Khan S *et al.* confirmed that extracellular survivin was associated with HPV infection in HPV-18 positive Hela cells (Khan et al., 2009). 2 years after that, the same authors discovered that extracellular survivin does exist in the exosomes (Khan et al., 2011).

As a kind of small non-coding RNAs, miRNAs can modulate the three prime untranslated region on the target mRNAs by the method of incomplete sequence pairing, thereby upregulating the expression of genes at a posttranscriptional level. miRNAs take part in many physiological and pathological processes, *via* different expression profiles and the interaction with inflammatory factors (Davidson-Moncada et al., 2010). Thus, it is not strange that dysfunctional miRNAs can lead to wide-spectrum human diseases, including malignancies, cardiovascular diseases, autoimmune diseases, neurological diseases, and so on (Alsaweed et al., 2015). When the cervical tissue gets malignant, several miRNAs (including lethal 7a, let-7a, miR-34a) have been discovered to be downregulated markedly and can lead to inflammation by upregulating their target genes. For example, let-7a has been identified to regulate STAT3 (signal transducer and activator of transcription) level, which plays an important role in connecting inflammation and cervical cancer. HPV oncoprotein E6 may directly or indirectly downregulate the expression of let-7a and promote the expression of STAT3, ultimately causing cervical carcinogenesis (Shishodia et al., 2015). Another example is about miR-34a, Zhi-ming Zheng et al. came to the conclusion that cervical cancer tissues could express a reduced level of miR-34a in a experiment composed of three pairs of normal cervix and cervical cancer tissues which were age-mached and positive for a single genotype. The same authors also found that the expression of miR-34a dependes on a functional p53 and can be downregulated by high risk HPV oncopratein E6 (Wang et al., 2009b).

## Nanotechnology in HPV DNA Detection

The application of nanomaterials in the field of clinical diagnosis was first realized through gold nanoparticles (Au NPs) in the late 1990s (Zehbe et al., 1997). In which, among the 10 HPV-positive cervical cancer specimens, only two were positive under the method of polymerase chain reaction, while there were nine positive tests when employed with the CARD (catalyzed reporter deposition)-nanogold technique (Zehbe et al., 1997). Since then, the application of gold nanoparticles in virus detection has been further developed.

The application of gold nanoparticles in HPV detection can be presented in many forms, including photoelectric analysis, fluorescence analysis, electrical and colorimetric analysis, etc (Baek et al., 2008; Zhang et al., 2011). Photoelectric analysis can enhance the deposition of metallic silver on its surface by AuNPs. After a series of steps, the target DNA will finally be quantified as a corresponding electrical signal. For fluorescence analysis, the most efficient form to detect HPV DNA is the combination of AuNPs and a microfluidic bead-based array. Through this system, the target DNA can be transformed into different enhanced fluorescence signals according to its concentration. Similarly, electrical and colorimetric analysis can also get an ideal sensitivity of HPV DNA detection (Draz and Shafiee, 2018).

Besides, many new techniques for HPV detection have been developed. For instance, Au-NP-coated superparamagnetic nanomaghemite with thiolated oligodeoxyribonucleotides complementary have been developed. The oligodeoxyribonucleotides could combine with the DNA sequence of the E6 oncogene of HPV-16. Then the combined ssDNA of the virus might be recognized by antibodies labeled with HWRGWVC peptide-modified-CdTe quantum dots. As a proof-of-concept, the authors conducted a test of HPV DNA in serum samples from 8 head and neck squamous cell carcinomas patients who had gotten historical conformation. By traditional PCR test, E6 HPV-16 was tested in five samples, and the data from the biosensor assay were in high accordance with PCR results. Furthermore, the components used in the test could be mass-produced and saved at 4°C for a long time. Taken together, this technique is prospecting in clinical translation (Jimenez Jimenez et al., 2017). The interaction of heat-resistant double-stranded DNA (dsDNA)-binding protein (Sso7d) and Au NPs to detect HPV genes is getting more and more attention from researchers. For example, Sso7d-modified Au NPs have been constructed by Ju-Yi Mao et al. to detect HPV genes with a detection limit of 1 copy of gene in specimens. The sensitivity and specificity data of this special probe from clinical specimens of Pap smear (*n* = 52) are exciting, as high as 85.7%/100% and 85.7%/91.7%, respectively (Mao et al., 2017). In addition to the application of the AuNPs, alone or combined with other techniques, a hybridization assay composed of quantum dots and superparamagnetic nanoparticle in detecting HPV 16 infections, or the mass spectrometry genotyping of HPV based on high-efficiency selective enrichment of nanoparticles, are both proved to be feasible than traditional methods (Yu-Hong et al., 2011; Zhu et al., 2018b). The exciting results also happened in other types of HPV, for instance, Maria D.L. Oliveira et al. designed a complex, consisting of a matrix of polyaniline (PANI) containing AuNPs, to help recognize different HPV types, including types 6, 11, 16, 31, 33, 45, and 58 (Avelino et al., 2020).

## Nanotechnology Associated With Neutralization by HPV Vaccine

The current vaccines (Gardasil® and Cervarix® et al.) available for HPV infection are comprised of virus-like particles (VLP) made of the type-specific major capsid protein L1, have little protection capacity against viral types which have no epitopes on the vaccine scaffold (Muñoz et al., 2004; Schiller and Müller, 2015). Furthermore, both of the L1-VLP HPV vaccines are intolerant to a higher temperature, which poses challenges for the preservation and transportation of vaccines (Shank-Retzlaff et al., 2006). In response to these deficiencies, vaccines targeting the minor capsid protein L2 of the HPV are appearing as widely protective alternatives for the current types (Schiller and Müller, 2015; Schellenbacher et al., 2017). In order to enhance the immunogenicity of the L2-VLP HPV vaccine, different methods have been put forward recently, among which, Thioredoxin-Displayed Multipeptide Immunogen L2 vaccine, based on thioredoxin from the hyperthermophilic archaeon Pyrococcus furiosus (PfTrx), which can provide ideal sites for multiple copies of peptide epitopes, has come into the spotlight (Canali et al., 2014; Seitz et al., 2015). For example, Gloria Spagnoli *et al* designed a kind of L2-VLP vaccine’s epitope and got a further enhancement of cross-neutralization capability by a multiepitope single-molecule formulation. The as-prepared PfTrx-L2 includes seven peptide epitopes high-risk HPV types and one L2 epitope from low-risk HPV6 type, as well as a self-assembling polypeptide OVX313. This cross-neutralization capability refers to that the formulation can elicit neutralizing antibodies against 10 different HPVs just with 8 viral types existing in the vaccine (Spagnoli et al., 2017). Further research showed that the existence of the OVX313 module could remarkably increase the anti-HPV16 neutralization titers in C57BL/6 mice. As a strong immunogen based on the minor capsid protein L2 of the HPV, PfTrx-OVX313 nanoparticles’ safety needs to be verified by more studies, especially by the prospective ones (Spagnoli et al., 2017).

## Nanotechnology in the Treatment of HPV Infection

Vaccines are developed to protect healthy people from HPVs infection by neutralizing antibodies, but for those who have been infected with HPVs already, there is no agreement on whether the vaccines can play a role in getting rid of the existed HPVs so far. Several therapies like cytotoxic agents, antiviral agents, immune modulators, cryotherapy, and CO_2_ laser therapy, mainly target benign, proliferative lesions. Besides, these therapies have no elicit effect and can be used only after the lesions have formed. In response to these shortcomings, HPV-based E6 and E7 oncoproteins, two key factors that can promote the abnormal proliferation of epithelial cells in the cervix, eventually arouse more and more attention in gene therapy (Münger et al., 2001; Moody and Laimins, 2010). Gene therapy refers to interfering with the expression of the E6/E7 gene of HPV by genome-editing systems transported by either viral vectors or non-viral vectors (Weber and Fussenegger, 2006). However, naked genes are unstable and unable to penetrate the cell membrane barriers. Therefore, a proper gene delivery system is needed to assist the intracellular transfection of a gene.

For example, Xueqin Gao et al. conjugated poly (amide-amine) (PAMAM, G0) with poly (β-amino ester) (PBAE) together to afford the hyperbranched copolymer PAMAM-PBAE (hPPCs). The resulting hPPCs were used to form a polyionic complex with CRISPR/Cas9 plasmid that targets the HPV16 E7. hPPCs significantly enhanced the cellular uptake of plasmid, escape of plasmid from endolysosome entrapment, and the subsequent gene editing efficiency (Guerrero-Cázares et al., 2014; Gao et al., 2020). Similarly, HPV16 E7-targeting CRISPR/short hairpin RNA was condensed by PBAE, the resulting nanocomplexes could not only downregulate HPV16 E7 expression, inhibit the growth of xenografts in nude mice, but also reversed the malignant cervical epithelium phenotype of HPV16 transgenic mice (Zhu et al., 2018a). Apart from polymers, cationic lipid nanoparticles (LNPs) are also efficient in gene delivery for the treatment of HPV-associated diseases. For example, small interfering RNA against HPV E6/E7 oncoproteins was encapsulated in LNPs composed of various lipids. Besides, the surface of LNPs was modified with epidermal growth factor receptor antibodies that can target cancer cells. Taken together, the combination of small interfering RNA against HPV E6/E7 and epidermal growth factor receptor antibodies significantly suppress the growth of xenograft tumors in mice (Kampel et al., 2021).

Moreover, the combination of chemotherapy and gene therapy is another promising strategy to combat HPV-induced malignancies. For example, Di et al. constructed a biomimetic dual-drug delivery system (Si/PNPs@HeLa) to deliver both paclitaxel and siRNA targeting E7. With the help of HeLa cell membranes camouflage, the system could avoid immune surveillance and accumulate on the tumor region, hindering the growth of tumor tissue successfully (inhibiting rates of 83.6% by tumor volume). Besides, the researchers also concluded that HeLa cells resistant to paclitaxel could be resensitized by knocking down the E7 gene (Xu et al., 2020).

## Immunotherapy

Apart from the preventative L1-VLP or possible L2-VLP vaccines mentioned above, therapeutic vaccines that promote antigen-specific humoral immune response are under development and will gradually be put into practice. In the development of therapeutic vaccines, it is important to choose an appropriate adjuvant. Recently, Liang *et al* showed that the engineered aluminum hydroxyphosphate nanoparticles (AAHPs) could be used to enhance antigen-specific antibody responses induced by HPV18 L1-VLPs by adjusting their surface charge. After a series of experiments, the researchers concluded that the positively charged AAHPs provoked much stronger and long-lasting antigen-specific humoral immune responses compared to their negatively charged or neutral counterparts (Liang et al., 2021). As an adjuvant of synthetic long peptides (from HPV16 E6 and E7 oncoproteins), mineral oil can help to form a persistent release depot but with severe adverse and long-lasting side effects. To address that problem, polymeric nanoparticles were used as a substitute for mineral oil. For example, poly (D,L-lactic-*co*-hydroxymethyl glycolic acid)-based delivery systems were developed for delivering the synthetic long peptide and a toll-like receptor 3 ligand. Similar therapeutic efficacy was observed in polymeric NPs and mineral oil formulations. The most important is that there is no adverse effect in the application of polymeric NPs containing poly IC (Rahimian et al., 2015).

## Conclusion

As an emerging technology, the application of nanotechnology in HPV infection is receiving increasingly more attention. Nowadays, most patients come to clinics or hospitals only after lesions have been discovered, such as verrucous lesions, cervical intraepithelial neoplasia, or even cancers. Therefore, it is very important to strengthen the publicity and education of relevant groups. As for HPV detection, the testing work in most developed countries is much better than that in underdeveloped countries and regions. Therefore, there is an urgent need to develop methods of high efficacy, low cost, ease to store, and transport. Our review has listed several nanomaterials, like Sso7d-modified Au NPs. Although the preclinical experimental results were exciting, a larger sample and further study in clinical forms are needed. For the treatment of HPV infection, the HPV vaccine neutralizing antibody is indeed a good choice. The latest method is to exploit the cross-antibody-neutralization effect to amplify the range of HPV types that vaccines work on, such as by the way of constructing a multiepitope single-molecule formulation. Apart from antibody neutralization of HPV vaccines by redesigning epitopes or changing types of adjuvants, gene therapy alone or in combination with chemotherapy are also efficient strategies to treat HPV-induced diseases. Of note, it is of great importance to perform repeated experimental verifications and consider safety, cost-effectiveness, and technical difficulties in choosing proper treatment for HPV-associated diseases.

## Abbreviations

AAHPs, the engineered aluminum hydroxyphosphate nanoparticles; Au NPs, gold nanoparticles; CRISPR, clustered regularly interspaced short palindromic repeats; hPPCs, the hyperbranched copolymer PAMAM-PBAE; HPV, human papillomavirus; let-7a, lethal 7a; LNPs, lipid nanoparticles; miRNAs, micro ribonucleic acids; PBAE, poly (β-amino ester); PCR, polymerase chain reaction; PfTrx, the hyperthermophilic archaeon Pyrococcus furiosus; STAT, signal transducer and activator of transcription; VLP, virus-like particles.
